# Caring for persons with Dementia: a qualitative study of the needs of carers following care recipient discharge from hospital

**DOI:** 10.1186/s12904-023-01322-1

**Published:** 2023-12-13

**Authors:** Janice Du Preez, Antonio Celenza, Christopher Etherton-Beer, Paula Moffat, Elissa Campbell, Glenn Arendts

**Affiliations:** 1 Department of Emergency Medicine, Fiona Stanley Hospital, Murdoch, Western Australia; 2 Emergency Department, Sir Charles Gairdner Hospital, Nedlands, Western Australia; 3 School of Medicine, University of Western Australia and Department of Geriatric Medicine, Royal Perth Hospital, Perth, Western Australia; 4 Palliative Care Unit, Bethesda Hospital, Claremont, Western Australia; 5 Department of Geriatric Acute and Rehabilitation Medicine, Sir Charles Gairdner Hospital, Nedlands, Western Australia; 6 Division of Emergency Medicine, School of Medicine, University of Western Australia, 35 Stirling Hwy, Crawley, WA 6009 Australia

**Keywords:** Dementia, End of life, Needs assessment, Carers

## Abstract

**Background:**

A randomised clinical trial titled the *Carer End of Life Planning Intervention (CELPI) in people dying with dementia* evaluated the effect of carer education and support about palliative care on care recipient outcomes. We present a pre-planned qualitative analysis of data collected during the CELPI trial in which needs of carers randomised to the study intervention group were assessed using a novel instrument (Carer Needs Directed Assessment in Dementia (CANDID). This tool aimed to identify carers’ perceptions of their own and their care-recipients’ needs and is an important step in identifying support provision for dementia-specific, palliative cares services upon hospital discharge.

**Methods:**

The CANDID tool was designed to identify the needs and experiences of primary carers and of their care recipients during the last twelve months of the care recipient’s life. The tool consisted of 33 open-ended questions evaluating: symptom management, emergency contacts, advance care planning, carer’s perception of the care recipient’s future needs, carer’s current needs, and a proposed current and future care plan. The researcher’s philosophical assumption of interpretative phenomenology informed the study and approach to data collection and analysis. Qualitative data collected during interviews using this tool were thematically analysed in five steps: compiling, disassembling, reassembling, interpreting and concluding. An interpretation of participants’ reality emerged from their common experiences and the subjective meanings assigned to actions attached to the phenomena studied.

**Results:**

Thirty carer participants were included. Analysis identified three major themes: Carers’ perceived stressors, systemic barriers to care provision, and future planning. Issues identified included barriers to accessing supports, carer health and division between roles, financial burden, familial conflicts, adquate care in hospital and aged care facilities, concern about future needs, and end-of-life discussions.

**Conclusion:**

The CANDID tool enabled an evaluation of carer needs and concerns. Identifying those needs may inform a referral to palliative care services where the level of management required may be benenficial for both the person living with dementia and their primary carer.

**Trial Registration:**

Australian Clinical Trials Registration: **(ACTRN12619001187134)**.

**Supplementary Information:**

The online version contains supplementary material available at 10.1186/s12904-023-01322-1.

## Background

In 2021, an estimated 1.6 million Australians cared for someone living with dementia [1]. Carers face increasing stressors as progressive cognitive difficulties and functional impairment in the person with dementia presents unique challenges for end-of-life care [2]. Evidence suggests that carers of people living with dementia prefer their care recipient to age-in-place i.e., in their own homes, with the support of palliative care services [3]. However, carers remain reliant upon emergency departments (EDs) to manage problems such as delirium, behavioural symptoms, or dysphagia [4]. With 95% of people with advance dementia requiring 24-hour care at end of life, many people are unable to die at home due to complex care needs and barriers to accessing palliative care services [5].

A knowledge gap about the terminal phase and end-of-life-care in dementia is shared by health professionals, primary carers, care staff and the community. Barriers to timely access of palliative services may include a lack of understanding on the part of primary carers of the disease trajectory [6]. A recent Cochrane review from 2016 concluded “that there is insufficient evidence to assess the effect of palliative care interventions in advanced dementia” [7]. Whilst palliative care may assist people with dementia and their carers, and can achieves outcomes that align with carer needs, it is rarely made available or accessed [8]. An understanding of the contributing factors driving reliance on EDs and the systemic barriers that inhibit access to services is needed. There is a paucity of research that identifies the needs of carers and their perceptions of their care recipient’s needs upon hospital discharge. This study aimed to identify carers’ perceptions of their own and their care-recipients’ needs that lead to ED care use and is an important step towards increased support provision for dementia-specific, carer needs directed, palliative cares services upon hospital discharge [9].

A randomised clinical trial that commenced in July 2019 titled the *Carer End of Life Planning Intervention (CELPI) in people dying with dementia* is ongoing, having commenced as a pilot trial that informed a larger nationwide study. This research is a pre-planned qualitative analysis of data collected using a new instrument *Carer Needs Directed Assessment in Dementia* (CANDID) [10] from carers randomised to the intervention arm of that trial. Perceived current and future needs of the primary carers and that of their care recipient’s life are evaluated. This tool aimed to identify carers’ perceptions of their own and their care-recipients’ needs and is an important step in identifying support provision for dementia-specific, palliative care services upon hospital discharge.

## Methods

The CANDID tool was developed for the purposes of the trial to identify the needs of the carers and their care recipients to plan provision of care. Development of the tool and an assessment of face, content, and criterion validity is described in detail elsewhere [10].

The trigger for carer inclusion in the study included an attendance to an ED by a care recipient with a diagnosis of severe dementia (as either a new or existing diagnosis), and an expected life expectancy of less than twelve months. Clinical records were screened and if the inclusion criteria were confirmed, primary carers were identified and contacted by recruiting clinicians for consent to participate in the study. Following randomisation, the participants in the intervention group had interviews with the intervention clinician which included the use of the CANDID tool.

Eligibility criteria for the person with dementia needed to be met that provided evidence for them having advance stage dementia and qualified the carer participants for this qualitative study.

These include:

Inclusion Criteria. The person with dementia must have:

1. Established prior documented diagnosis of dementia, supported by an appropriate assessment screen e.g., Mini Mental State Exam (MMSE) < 13/30 [11] or a Montreal Cognitive Assessment (MoCA) < 16/30 [12].
2. Functional Assessment Staging Test (FAST) Stage 6d-7f [13].

### The CANDID tool

The tool consisted of 33 open-ended questions evaluating: symptom assessment, emergency contacts, advance care planning, carer’s perception of the care recipient’s future needs, carer’s current needs and a proposed current and future care plan. The tool, a novel component, assisted in thorough identification of the perceived needs of these carers for providing comprehensive care [14, 15]. The tool assessed:

- **Carer’s perception of their care recipient’s current needs**: Is the carer aware of the stages of dementia and are they aware their care recipient has advance-stage dementia?
- **Symptoms and symptom management**: Is the carer able to determine whether their care recipient is in pain, is properly hydrated, having adequate nutrition? Are they able to manage these symptoms or report them to their current care service providers in a clear and timely fashion?
- **Advance care planning**: Is there a signed Advance Care Directive or an Advance Care Plan in place that includes an allocated power of attorney or guardianship, a goals of care statement including use of resuscitation and other wishes as stated by the care recipient or by carer proxy?
- **End of life discussion**: Has the carer been informed about their carer recipient being deemed at end of life and do they understand what the process of dying looks like?
- **Spirituality and potential end of life rites and rituals**: Has the care recipient’s wishes both culturally and spiritually been noted and plans made for example, the order of a funeral service, to ensure these wishes will be executed at end of life?
- **Equipment provision**: Should the care recipients wish to be cared for in their home, has provision been made for the necessary equipment for example a floor line bed, a hoist, crash mats, and a shower commode to promote the care recipient’s safety, pressure management and proper manual handling by the carer and support staff?
- **Carer’s current and future needs**: Has the carer made provision for their care recipient’s future care needs and their own upon the recipient’s death?

The CANDID tool provided a general framework and facilitated consistency across semi-structured interviews which were approximately 2.5 h in duration and conducted in participant’s homes. The CANDID tool maintained a focus on the experiences, and current and future perceived needs of participants and their care recipients upon hospital discharge. Participants were encouraged to describe their experiences on issues outside of the CANDID interview schedule in keeping with qualitative research principals. Questions aimed to elicit responses about their experiences in the home and during residential aged care admission.

Primary carers were recruited following a visit by their care recipient to an ED. Different data collection methods were employed which include: the researcher screening patient clinical notes, cognitive assessment outcomes, baseline data to verify and establish current needs and anticipated/perceived future need, and to confirm participant interview data.

The tool facilitated collaboration with the carer and intervention clinician of the study to plan, support and resource for the care receipient’s current and future needs. Where deemed appropriate, and with the permission of the carer, a referral was made to a specialist palliative care service via the care reciepient’s General Practitioner.

In-context positioning and the use of methodologies that elicited a narrative using sources such as in-context observation, in-depth interviews, and researcher fieldwork journal reflections generated knowledge [16, 17]. This approach aimed to elicit the personal perceptions, experiences and needs of carers and their care recipients upon discharge from hospital to their homes or to a residential aged care facility [14, 15]. An interpretation of the participant’s reality emerged from their shared experiences and the subjective meanings assigned to actions attached to the phenomena studied during data collection and analysis [16, 18, 19]. By regarding the participants as the experts on their lived experiences, the researcher was able to define the stressors and barriers participants viewed as critical.

Exploring the meanings and contexts of people’s daily lives, their occupations and their experiences enabled the researcher to understand how the reality of caring was experienced by those studied [20, 21]. Thick descriptions of participant experiences are substantiated by verbatim quotes [21–23]. Additional use of considered commonalities across interviews such as, participant’s feelings about service provision and their concerns about the level of end of life care, should something happen to them, culminated in a synthesis of findings adding rigor to this approach [16–18, 24, 25].

### Rigour

With a view to minimizing the risk of bias and maximise the accuracy of research results rigour was established and achieved by deliberate and careful planning, maintenance of an audit trail and fieldwork reflexivity on the part of the researcher [26]. To further ensure trustworthiness of the qualitative research, the researcher applied four criteria [24].

#### Credibility

Prolonged engagement with data whereby the researcher immersed themselves in the context of the study, building trust with each participant and overcoming distortions due to their presence (cf., Hawthorne Effect) [19].

#### Dependability

Dependability of findings was achieved through the researcher’s methodical description of process decisions [27].

#### Confirmability

By using verbatim participant quotes, the researcher confirmed that findings were elicited from the data and not from the researcher’s personal biases [24].

#### Transferability

Using thick descriptions to describe the findings, the researcher has ensured that the methodology can be transferred to other contexts with other respondents [24].

### Data collection and analysis

To ensure data collection and analysis was an iterative process, interviews were manually recorded and transcribed verbatim immediately after each interview [16]. Saturation was reached when participant interviews reached information sufficiency, i.e., “where evidentiary value depends on the rigor of the analytical process (analytical sufficiency) and the richness of data (data sufficiency) generated during interviews” [28].

Breaking down the qualitative dataset into smaller samples, the author used open coding creating codes based on the qualitative data itself. Codes arose directly from the participants responses to the interview questions. The sample was re-read and the codes applied. Each new sample of data was read and the initial codes created were applied. Where the codes did not match, the author embraced qualitative research values and utilised subjective skills to reflect upon and document thoughts and the differences and reasons for creating additional codes. This interpretative reflexive process did not require a coding framework and the iterative method of inductive coding ensured unbiased, reliable, consistent, accurate coding and a complete look at themes through the data [29].

The researcher used a combination of manual coding and computer assisted qualitative data analysis software, QSR NVivo 11 [30], to code, categorise and analyse data and themes, and from these recommendations emerged [31, 32]. Using a thematic analysis framework, the researcher coded the transcripts to organize and assemble the data collected into recurring topics. The code list developed from the list of research questions contained within the CANDID. Other items considered during coding and categorising include: the setting/context within which the carer’s experience took place, how they defined the setting, participant perspectives, specific activities or events, strategies used by participants to accomplish things, relationships and social structure of the carer and their care recipient to the setting, person, or organisation. Once data was coded, the data was categorised and hypothesis construction developed as emergent themes i.e., the grouping of one or more categories clustered around an inductively derived central concept became evident [33]. Investigator team members reviewed proposed themes to determine how well they fit the data. Themes were revised to ensure conceptual clarity. Nine categories describing barriers and facilitators were identified, referring to three global themes: Aspects related to (1) carer health and burden (2) systemic barriers (3) end-of-life discussion.

## Results

Of the thirty-three participants randomised to the intervention group, thirty were interviewed over a two-year period with each interview lasting approximately 2.5 h in duration. The interviews were conducted between September 2019 and April 2021. An occupational therapy practitioner/PhD qualified qualitative researcher and the first author listed conducted the interviews and did the initial coding. Two care recipients died before the intervention could be conducted, and one participant could not be contacted.

Ten of the participants interviewed were spousal carers with three being husbands, and seven being wives. Eleven were daughters, six were sons, three were other relatives and one was a close friend. The participants ranged in age from 52 to 88 years old and included 13 males (mean age 68.5 years) and 17 females (mean age 73.6 years). Eight care recipients resided in their own homes with their primary carer and twenty-two resided in residential aged care facilities.

Analysis of participant’s responses were distilled into three major themes. Three main themes were identified: Carers’ perceived stressors, systemic barriers to care provision, and future planning. A detailed exposition of these themes is described using anonymised quotes with fictitious participant numbers for the participants themselves. Throughout, the voices of the participants are heard as a way of validating their experiences.

### Theme one: carer’s perceived stessors

*“I am too busy to worry about my own health at the moment.”* P006

### Carer health

Participants described how their caregiving role was compounded by their own complex care needs which included cancers, strokes, and knee and back surgeries that make lifting and transferring their care recipients difficult and make caregiving challenging.

*“MRI’s have revealed multiple strokes… and an aneurysm which is affecting my balance…”* (P037)

*“I tried a hoist, but this place is too small for it. So, I lift her myself.”* (P045)

Stressors such as physical pain, grief and fear of their own mortality being imminent resulted in participants experiencing mental illness such as depression and anxiety soothed through self-medication and alcohol abuse *“I smoke and drink too much”* (P013) and “*I have anxiety and insomnia. I have a lot to worry about… and I have digestive problems”* (P016). Some participants reported being hospitalised after multiple suicide attempts.

### Loss and grief

Some participants described their guilt, grief and ongoing sense of reponsibility after admitting their care recipient to a residential aged care facility, “*I worry about what will happen if anything happens to me. I feel guilty that I couldn’t continue to care for him in his home…It was just terrible leaving him there.”* (P024) *“I have been struggling. There are days that are very dark, and I feel lost. I am on anti-depressants, but I can’t be bothered.*” (P055)

### Multiple caring roles

Participants reported feeling ‘sandwiched’ between caring for their care recipients in their own homes i.e., in a home owned by the care recipient but not one in which the participants lived. For example, for one participant the role of caregiver was multi-faceted involving her rising at 6am to meet the needs of her immediate family, travelling to her care recipient’s home to address their personal care and feeding needs, then travelling to another home to care for her autistic grandson. At 4pm, once her daughter had returned home from work, she would reverse the process, ensuring her mother had been fed and *“locked all the doors and windows and leave her there, alone, for the night” (P006)* until she could return in the morning. Despite the toll this took on her own health, the decision to admit her care recipient to a residential aged care facility was filled with guilt that continues to this day. Other participants reported caring for both parents with dementia simultaneously sacrificing relationships to do so.

*“I feel “sandwiched” between my children and my mother when really all I want to do is move over east to the acreage my boyfriend lives on.” (P007)*

### Familial support

#### Conflicts

within familial relationships was common among participants. Some participants stoically refused to ask family for help, insisting they had *“enough to do,”* while others reported that their pleas for help, or even a moment’s respite, was ignored as siblings deflected and avoided assuming any caring responsibilities. When asked by the researcher whether the participants felt supported by their family they replied, “… *they have their own things to worry about”* (P046) and *“…none of them have offered to care for her, not even for a weekend.”* (P022) Another participant said,

*“My brother has washed his hands of my mother and doesn’t want anything* *to do with this.” (*P008)

One participant became very emotional as she considered her answer then described feeling guilt and condemnation from family who live abroad, and strongly oppose their mother’s admission to a residential aged care facility. Several participants expressed gratitude for the time they get to spend with their care recipients. Here a participant describes how visiting her parents, who share a room in a residential aged care facility has provided surprising benefits,

“*Their faces light up when I visit. Now, after all those years of ‘cold, emotionless’ parenting, Mum gets very emotional, and they both hug me all the time. It’s lovely.”* (P028)

### Financial burden

The cost of housing concerned those participants who did not own their home and who are unable to access social housing. Participants expressed their anguish about their housing situation stating,

*“I do get a carers allowance but that doesn’t take you very far. If she dies, I have nowhere to go.”* (P045)

*“…once Mum goes, I will lose my carers allowance and I have nothing to live on. I have no pension and neither does my mum.”* (P052)

Carer’s inability to comprehend the complexities of the aged care system, coupled with computer illiteracy meant that they were unable to access services that could provide them with the information and support they required.

*“I don’t understand this system. The problem is I do not know who to talk to…”* (P002)

### Theme two: systemic barriers to care provision

*“I don’t like the idea of her dying but I don’t like the way she is living either. This is not how she would have chosen to live.”* (P007)

### Hospital admissions and residential aged care services

The lack of attention given by hospital staff to patients’ past medical histories, dietary considerations, toileting regimes, medical prescriptions, pressure injuries sustained from non-adherence to proper turning protocols, lack of communication between service providers, lack of appropriate, dementia-specific signage, and the disregard for carer stressors when transferring their care recipient to multiple wards, to a different hospital or to an undisclosed transitional care facility, were all issues that participants found unbearable and overwhelming. These issues are highlighted in this statement,

*“One UTI (*urinary tract infection*) left her ‘comatose’, and she was hospitalised. They were unable to catheterise her to obtain a urine sample, so she was transferred from ED to another ward, then another ward until they told me she was being transferred to another hospital. The doctor was supposed to meet me at the hospital at 4.30pm that afternoon but she was playing golf instead. I had spoken to every nurse on every ward at both hospitals regarding her special diet, but they ignored me. I took a can of supplement to the hospital to ensure she was getting something. She developed bed sores and as they really couldn’t do anything for her, I insisted they bring her back home.”* (P040)

A lack of dementia-specific trained hospital staff was evident to participants as one succinctly explained,

*“People with advance stage dementia are unaware they have the condition. Asking them to provide their past medical history is not good practice. Also, asking their carers certain questions in front of them can create anxiety and a ‘melt-down’ by the patient. Hospital staff need training on how to overcome these barriers.” (P028)*.

Concerns about residential aged care providers were raised by most participants whose care recipients were housed in a facility. Under-staffing and lack of training in dementia-specific care were the main areas of concern expressed. Examples of lack of proper care provision were reported,

*“The carers are too busy. Dad was initially very resistant to showering… the staff didn’t take control of the situation, so he stayed in a full continence pad for three days.”* (P024)

*“I have visited him on occasion and found him sopping wet from urine. The staff only clean his room once a week but given that he is using the whole room as a toilet, I want them to clean it at least every second day.”* (P037)

Symptom mismanagement such as pain management, implementing dysphagia protocols, nutrition and hydration, toileting regimes, continence management, managing changed behaviours, use of restraint and falls prevention strategies within hospital and residential aged care settings were areas of particular concern raised by participants. Poor implementation of protocols, infrequent monitoring and charting, and inconsistent evaluation in symptom management caused increased stress for participants who had entrusted their care recipients into care. The impact of mismanagement is described in the following participant quote,

*“Upon hospital discharge* [with a neck of femur repair] *at first the residential aged care facility didn’t want to take him home and the hospital wanted the bed. That was very stressful. But once back at the residential aged care facility, he was left in his bed to recover…the Facility Manager said, ‘There are 90 residents, and the physiotherapist can’t get to them all’.* (P063)

### Theme three: future planning

“*I am terrified of that. How will I know when it’s the end?* (P025)

### End of life discussion

Regarding advance care planning and end of life discussion, participant comments ranged from doctors who avoided end of life discussions *“the doctor gave me a brochure ‘What to do when someone dies’* (P045) and comments about their fear of death, and their own expectations,

“*We have not signed a Do Not Resuscitate order as we want the doctors to “do their best” to keep her alive.”* (P042).

“*No. I don’t want to talk about it. I would rather not know and just take it as it comes”* (P059).

*“I don’t want to know. I can’t face thinking about him choking or struggling for breath.”* (P030).

### Perceived future needs

The greatest stressor and aspect of caring for their care recipient was the anxiety about their own mortality and fear about who would care for their care recipient should something happen to them. With some participants facing a terminal diagnosis, anguish about the level of care their care recipient would receive without them [the participant] to advocate on their behalf was conveyed, *“I do worry about what would happen to them if I died. Would they be properly cared for?* (P063) *and “I am worried that something happens to me, and I won’t be able to be there for her.”* (P001).

## Discussion

In this study we report comprehensive data on the needs of primary carers of people living with dementia, informed by a new carer-directed needs assessment designed for a clinical trial. This study emphasizes carers’ perceived needs, the barriers hindering access to accessible resources and inadequate models of care across service providers that drive ED admissions rather than supporting access to palliative care services.

While evidence suggests that carers of people living with dementia prefer their care recipients to age-in-place i.e., in their own homes, those with advanced dementia who require 24-hour care at end of life are unable to die at home due to complex care needs and barriers to accessing palliative care services [3, 5]. Additionally, apparent poor service provision and low levels of dementia-specific training of hospital staff, in-home care service providers and residential aged care providers leaves carers feeling overwhelmed by circumstances beyond their control resulting in them having anxiety, depression, suicidal ideation, and mental ill health. This is congruent with findings by Diwan, Hougham and Sach (2004) and Robinson et al., (2014) who found that severe cognitive difficulties in the person with dementia increase carer stressors and present unique challenges for end-of-life care.

Family carers often fail to recognise that they are no longer able meet their carer recipient’s needs [34]. Watching others assume responsibility for caring for their care recipient is a difficult transition with carers remaining vigilant of the level of care being provided, torn between needing to ensure their care recipient enjoys the same level of care they were able to provide, and wanting to enjoy their freedom. Some become indifferent and withdraw, reluctant to assume responsibility for decision-making regarding their medical care or advocating on their care recipient’s behalf leaving the caring role and decision making to service providers [34].

Communication between primary carers, family and multidisciplinary hospital and residential aged care staff is often poor, leading to depersonalised care for people with dementia, including palliative care [35]. Carers’ lack of knowledge, stoicism, attitudes and understanding of palliative care often serve as barriers to them accessing formal support which is rather avoided for as long as possible. Any discussion about coming to terms with and understanding what a palliative care referral means and what the dying process comprises is often found to be abhorrent and something to be avoided.

Many reactive hospital admissions could potentially be avoided if the issues commonly described by participants were better managed by health professionals and earlier referral to palliative care support [4, 36]. Predicting death trajectory timeframes makes prognostication in dementia a complex task for clinicians [37, 38]. Evidence suggests that clinicians’ reluctance to predict palliation and the need for end-of-life care is one of the main barriers to accessing specialist palliative care services [39].

When communication between stakeholders is timely, appropriate, collaborative, and person-centred, care provision can ensure quality end-of-life care [40]. A critical literature review that explored ‘the challenges of delivering effective palliative care to older people with dementia in the acute hospital setting’ conducted by Birch et al., (2008) is consistent with the findings in this current study. When the collective intention is to promote quality of life, minimise hospital admission and length of stay through the timely provision of specialist palliative care services, people dying of dementia will “*no longer be subjected to protracted, potentially uncomfortable and undignified deaths*.” [41].

### Implications for practice

This data supports the use of the CANDID tool in practice. The tool elicits carers’ perceptions about their care recipient’s current and future needs enabling clinicians to understand where additional support is required and to work collaboratively with carers to establish the goals of care and future care planning. A palliative care approach for people living with dementia refers to inclusive care that includes treatment of their health issues, comorbidities and behavioural and psychological symptoms of dementia. This approach forms the baseline level of care however, a continuum of care from the point of diagnosis throughout the disease trajectory with specialist palliative care addressing changing care goals should be available [42].

Health professionals may need to assist carers to navigate changes in social and peer interactions, familial relations, intimate relationships, spiritual attitudes, a complex aged-care system, and diverse levels of service provision. Facilitating and enabling adaptation and acceptance of disease progression, palliative care, end-of-life issues, and symptom management by the caregiver falls to the health professionals who are caring for them. Having those difficult conversations and knowing how to collaboratively find solutions that facilitate positive change is essential to minimize distress associated with end-of-life caregiving.

### Study-strengths

The CANDID tool encouraged the participants to speak at length and to highlight the concerns that were critically important to them. Data was collected from participants who are not regularly heard. Furthermore, use of open-ended questions that allowed participants to share rich content enabled identification of key aspects of their experiences.

### Limitations

Coding and categorising was completed by the researcher independently and not reviewed for consenus by the investigative team. However, as described, the coding process ensured unbiased, reliable and accurate coding.

## Conclusion

An assessment of carers’ service needs should include questions that elicit the primary carer’s perceptions of their levels of stress, anxiety or depression and whether or not the carer feels the need for additional support. The CANDID tool used in this study can identify detailed information about these factors and direct future management. Whilst there are services available to support carers in Australia such as information, support groups, respite care, education, psychosocial therapies and some programs that combine these components, barriers to accessing the supports remains an issue for carers [43]. A referral to palliative care services provides for this level of assessment and is therefore beneficial for both the person living with dementia and their primary carer. Additional policies that consider infrastructures that support carers, enabling them to care for themselves, and have the potential to improve their quality of life and health outcomes are recommended.

### Electronic supplementary material

Below is the link to the electronic supplementary material.

Supplementary Material 1

## Data Availability

Availability of the raw data is available from the corresponding author upon request.
