# Correction: Caring for persons with dementia: a qualitative study of the needs of carers following care recipient discharge from hospital

**DOI:** 10.1186/s12904-024-01362-1

**Published:** 2024-01-26

**Authors:** Janice Du Preez, Antonio Celenza, Christopher Etherton-Beer, Paula Moffat, Elissa Campbell, Glenn Arendts

**Affiliations:** 1 Department of Emergency Medicine, Fiona Stanley Hospital, Murdoch, Western Australia; 2 Emergency Department, Sir Charles Gairdner Hospital, Nedlands, Western Australia; 3 School of Medicine, Department of Geriatric Medicine, University of Western, Royal Perth Hospital, Perth, Western Australia; 4 Palliative Care Unit, Bethesda Hospital, Claremont, Western Australia; 5 Department of Geriatric Acute and Rehabilitation Medicine, Sir Charles Gairdner Hospital, Nedlands, Western Australia; 6 Division of Emergency Medicine, School of Medicine, University of Western Australia, 35 Stirling Hwy, 6009 Crawley, WA Australia

**Correction: BMC Palliat Care 22, 200 (2023)**

10.1186/s12904-023-01322-1

Following publication of the original article [1], the author reported that the given names and family names were switched. This has been corrected above and the original article has been updated.
