# Supplementary material for: Caring for persons with Dementia: a qualitative study of the needs of carers following care recipient discharge from hospital
Source: BMC Palliat Care. 2023 Dec 13;22:200. doi: 10.1186/s12904-023-01322-1 (PMC10717287; doi:10.1186/s12904-023-01322-1)
Supplement: Supplementary file 1 — Supplementary Material 1 [file 12904_2023_1322_MOESM1_ESM.docx]

**CARER-DIRECTED NEEDS ASSESSMENT IN DEMENTIA**

| NAME OF CARER | |  | | DOB |  | M | F | OTHER |
| --- | --- | --- | --- | --- | --- | --- | --- | --- |
| NAME OF CARE RECIPIENT | |  | | | | | | |
| RELATIONSHIP TO CARE RECIPIENT | |  | | | | | | |
| IN-HOME CARE ADDRESS | | | RESIDENTIAL AGED CARE FACILITY ADDRESS | | | | | |
|  | | |  | | | | | |
|  | | |  | | | | | |
|  | | |  | | | | | |
|  | | |  | | | | | |
| **EMAIL ADDRESS:** |  | | | | | | | |
| NAME OF INTERVENTION CLINICIAN | |  | | | | | | |
| DATE OF INTERVIEW | |  | | | | | | |
| DURATION OF INTERVIEW | |  | | | | | | |

This questionnaire has been developed to assist you in identifying your current and future care needs so that we can best support you. Together, we will determine what your care recipient’s and your needs are currently and what they may be in the future. Together you and I will design a care plan to ensure that you and your care recipient are supported throughout the dementia journey.

**CARERS PERCEPTION OF THEIR CARE RECIPIENT’S CURRENT NEEDS:**

- **Use this adapted/modified Symptom Assessment Scale (SAS) data to verify and establish current needs and anticipated/perceived future needs.**

| **Carer Rated Score** | **Symptom Assessment Scale (0-10)** *Rate experience of symptom distress over a 24hr period 0 = absent 10 = worst possible*  0 = Continue care 1 -3 = Monitor and record 4-7 = Review/change plan of care; referral, intervention as required 8-10 = Urgent action | | | | | | | | | | | | | | |
| --- | --- | --- | --- | --- | --- | --- | --- | --- | --- | --- | --- | --- | --- | --- | --- |
|  | Distress from difficulty sleeping |  |  |  |  |  |  |  |  |  |  |  |  |  |  |
|  | Distress from Appetite |  |  |  |  |  |  |  |  |  |  |  |  |  |  |
|  | Distress from Nausea |  |  |  |  |  |  |  |  |  |  |  |  |  |  |
|  | Distress from Bowels |  |  |  |  |  |  |  |  |  |  |  |  |  |  |
|  | Distress from Breathing |  |  |  |  |  |  |  |  |  |  |  |  |  |  |
|  | Distress from Fatigue |  |  |  |  |  |  |  |  |  |  |  |  |  |  |
|  | Distress from Pain |  |  |  |  |  |  |  |  |  |  |  |  |  |  |
|  | Other |  |  |  |  |  |  |  |  |  |  |  |  |  |  |
|  | **Rated by Carer** |  |  |  |  |  |  |  |  |  |  |  |  |  |  |

| **Problem Severity Score Actions (0-3)** *Refer to complete definition and rate each domain*  0 = Continue care 1 = Monitor and record 2 = Review/change plan of care; referral, intervention as required 3 = Urgent action | | | | | | | | | | | | | | |
| --- | --- | --- | --- | --- | --- | --- | --- | --- | --- | --- | --- | --- | --- | --- |
| Pain |  |  |  |  |  |  |  |  |  |  |  |  |  |  |
| Other Symptoms |  |  |  |  |  |  |  |  |  |  |  |  |  |  |
| Psychological / Spiritual |  |  |  |  |  |  |  |  |  |  |  |  |  |  |
| Family / Carer |  |  |  |  |  |  |  |  |  |  |  |  |  |  |

| **Palliative Care Phase**  **Stable =** Monitor **Unstable =** Urgent action required **Deteriorating =** Review plan of care **Terminal =** Provide EOL care |
| --- |

| **Problem Severity Score Actions (0-3)** *Refer to complete definition and rate each domain*  0 = Continue care 1 = Monitor and record 2 = Review/change plan of care; referral, intervention as required 3 = Urgent action | | | | | | | | | | | | | | |
| --- | --- | --- | --- | --- | --- | --- | --- | --- | --- | --- | --- | --- | --- | --- |
| Pain |  |  |  |  |  |  |  |  |  |  |  |  |  |  |
| Other Symptoms |  |  |  |  |  |  |  |  |  |  |  |  |  |  |
| Psychological / Spiritual |  |  |  |  |  |  |  |  |  |  |  |  |  |  |
| Family / Carer |  |  |  |  |  |  |  |  |  |  |  |  |  |  |

| **Palliative Care Phase**  **Stable =** Monitor **Unstable =** Urgent action required **Deteriorating =** Review plan of care **Terminal =** Provide EOL care |
| --- |

**ADVANCE CARE PLANNING**

| **Is there a documented Advance Care Plan?** |  |
| --- | --- |
| **Would you like to know more about an Advance Care Plan?** |  |
| **Are there any particular rituals he/she would like to see actioned as part of their death experience, after-death experience?** |  |

**CARER’S CURRENT NEEDS**

| **Are there any aspects of your care recipient’s disease that you do not understand or would like to know more about?** |  |
| --- | --- |
| **Do you have any concerns related to your own health today?** |  |
| **Are there any legal issues you need addressed?** |  |
| **Do you have any practical help/ services in the home?** |  |
| **Are you satisfied with the level of service provision being provided?** |  |

**EQUIPMENT NEEDS:**

**Is there any equipment you feel you need to assist you in caring for your care recipient?**

| **Shower chair** |  |
| --- | --- |
| **Detachable shower hose** |  |
| **Non-slip mat** |  |
| **Hoist** |  |
| **Portable commode** |  |
| **Over-toilet frame** |  |
| **Hand rails** |  |
| **OTHER:** |  |
|  |  |

**MIDDLE OF THE NIGHT EMERGENCY**

In an emergency, who would you call?

**Do you have contact numbers of various services that could provide you with help?**

| CONTACT’S NAME: | CONTACT NUMBER: |
| --- | --- |
| **General Practitioner most familiar with situation** |  |
| **Psychological Counsellor** |  |
| **Family member** |  |
| **Ambulance** |  |
| **Funeral director** |  |

**PROPOSED CURRENT CARE PLAN:**

| IDENTIFIED AREAS OF NEED: | ACTION TAKEN | REFERRAL MADE TO | DATE | REVIEW DATE |
| --- | --- | --- | --- | --- |
|  |  |  |  |  |
|  |  |  |  |  |
|  |  |  |  |  |
|  |  |  |  |  |
|  |  |  |  |  |
|  |  |  |  |  |
|  |  |  |  |  |

**PROPOSED FUTURE CARE PLAN:**

| IDENTIFIED POTENTIAL AREAS OF NEED: | ACTION | REFER TO | DATE | REVIEW  DATE |
| --- | --- | --- | --- | --- |
|  |  |  |  |  |
|  |  |  |  |  |
|  |  |  |  |  |
|  |  |  |  |  |
|  |  |  |  |  |
|  |  |  |  |  |
|  |  |  |  |  |

**ADDITIONAL NOTES:**
